# *N*,*N*′-Bis(3-nitro­benzyl­idene)-2,2′-[2-(3-nitro­phen­yl)imidazolidine-1,3-di­yl]diethanamine

**DOI:** 10.1107/S1600536810003168

**Published:** 2010-02-03

**Authors:** Mohammad Hossein Habibi, Narges Abarghooei-Shirazi, Yuki Yamane, Takayoshi Suzuki

**Affiliations:** a Catalysis Division, Department of Chemistry, University of Isfahan, Isfahan 81746-73441, Iran; b Department of Chemistry, Faculty of Science, Okayama University, Tsushima-naka 3-1-1, Okayama 700-8530, Japan

## Abstract

The title compound, C_27_H_27_N_7_O_6_, a Schiff base, was synthesized by the reaction of triethyl­enetetra­mine with 3-nitro­benzealdehyde. There are two independent mol­ecules in the asymmetric unit. The central aromatic ring in one mol­ecule makes dihedral angles of 23.99 (7) and 20.06 (6)° with the two terminal rings; for the second mol­ecule, these angles are 26.14 (6) and 24.64 (6)°.

## Related literature

For related structures, see: Glidewell *et al.* (2005 ▶, 2006 ▶); Habibi *et al.* (2007 ▶); Li *et al.* (2005 ▶). 
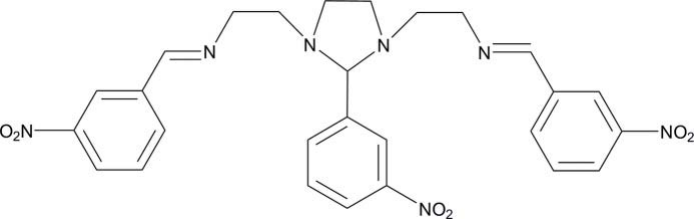


## Experimental

### 

#### Crystal data

C_27_H_27_N_7_O_6_
*M* *_r_* = 545.56
Monoclinic, 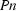

*a* = 13.3411 (12) Å
*b* = 10.4347 (8) Å
*c* = 19.8517 (17) Å
β = 103.446 (3)°
*V* = 2687.8 (4) Å^3^
*Z* = 4
Mo *K*α radiation
μ = 0.10 mm^−1^
*T* = 193 K
0.30 × 0.20 × 0.15 mm

#### Data collection

Rigaku R-AXIS RAPID diffractometer
Absorption correction: multi-scan (*ABSCOR*; Higashi, 1995 ▶) *T*_min_ = 0.971, *T*_max_ = 0.985
25846 measured reflections
6145 independent reflections
4184 reflections with *I* > 2σ(*I*)
*R*_int_ = 0.035

#### Refinement

*R*[*F* ^2^ > 2σ(*F* ^2^)] = 0.035
*wR*(*F* ^2^) = 0.100
*S* = 1.09
6145 reflections
722 parameters
2 restraints
H-atom parameters constrained
Δρ_max_ = 0.18 e Å^−3^
Δρ_min_ = −0.19 e Å^−3^

### 

Data collection: *PROCESS-AUTO* (Rigaku, 1998 ▶); cell refinement: *PROCESS-AUTO*; data reduction: *CrystalStructure* (Rigaku, 2004 ▶); program(s) used to solve structure: *SIR2004* (Burla *et al.*, 2005 ▶); program(s) used to refine structure: *SHELXL97* (Sheldrick, 2008 ▶); molecular graphics: *ORTEP-3 for Windows* (Farrugia, 1997 ▶); software used to prepare material for publication: *SHELXL97*.

## Supplementary Material

Crystal structure: contains datablocks I, global. DOI: 10.1107/S1600536810003168/bt5175sup1.cif

Structure factors: contains datablocks I. DOI: 10.1107/S1600536810003168/bt5175Isup2.hkl

Additional supplementary materials:  crystallographic information; 3D view; checkCIF report

## Figures and Tables

**Fig. 1.**
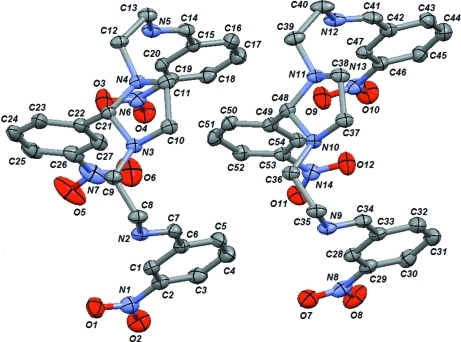
A view of the title compound, showing the atom-numbering scheme. Displacement ellipsoids are drawn at the 30% probability level and the hydrogen atoms are omitted for clarity.

## supplementary crystallographic information

### Comment

The design of Schiff-base complexes has received long-lasting research interest not only because of their attractive structural and topological novelty. Structures of Schiff bases derived from nitrobenzaldehydes and related to the title compound have been reported by Li *et al.* (2005), Glidewell *et al.* (2005, 2006), and Habibi *et al.* (2007).

The title compound (Fig. 1) was synthesized by the reaction of triethylenetetramine with 3-nitrobenzealdehyde, and its crystal structure is reported here.

The orientations of the C1–C6 and C15–C20 benzene rings respect to the C22–C27 ring are indicated by the dihedral angles of 23.90 (5) and 20.43 (5)°, respectively.

### Experimental

The title compound was synthesized by adding triethylenetetramine (0.146 g, 1 mmol) into a solution of 3-nitrobenzealdehyde (0.453 g, 3 mmol) in methanol (10 ml). The mixture was refluxed with stirring for 3 d. The resultant yellow solution was filtered. Pale yellow columnar single crystals of (I) were formed after slow evaporation of the solvent at room temperature (81% yield).

### Refinement

In the absence of anomalous scatterers, Friedel pairs were merged. Hydrogen atoms were refined using a riding model with C-H ranging from 0.95 to 1.00Å and U(H)=1.2U_eq_(C).

### Crystal data

**Table:**

C_27_H_27_N_7_O_6_	*F*(000) = 1144
*M**_r_* = 545.56	*D*_x_ = 1.348 Mg m^−^^3^
Monoclinic, *P**n*	Mo *K*α radiation, λ = 0.71075 Å
*a* = 13.3411 (12) Å	Cell parameters from 16587 reflections
*b* = 10.4347 (8) Å	θ = 3.0–27.5°
*c* = 19.8517 (17) Å	µ = 0.10 mm^−^^1^
β = 103.446 (3)°	*T* = 193 K
*V* = 2687.8 (4) Å^3^	Columnar, pale yellow
*Z* = 4	0.30 × 0.20 × 0.15 mm

### Data collection

**Table:**

Rigaku R-AXIS RAPID diffractometer	6145 independent reflections
Radiation source: fine-focus sealed tube	4184 reflections with *I* > 2σ(*I*)
graphite	*R*_int_ = 0.035
Detector resolution: 10.00 pixels mm^-1^	θ_max_ = 27.5°, θ_min_ = 3.0°
ω scans	*h* = −14→17
Absorption correction: multi-scan (*ABSCOR*; Higashi, 1995)	*k* = −13→13
*T*_min_ = 0.971, *T*_max_ = 0.985	*l* = −25→25
25846 measured reflections	

### Refinement

**Table:**

Refinement on *F*^2^	Primary atom site location: structure-invariant direct methods
Least-squares matrix: full	Secondary atom site location: difference Fourier map
*R*[*F*^2^ > 2σ(*F*^2^)] = 0.035	Hydrogen site location: inferred from neighbouring sites
*wR*(*F*^2^) = 0.100	H-atom parameters constrained
*S* = 1.09	*w* = 1/[σ^2^(*F*_o_^2^) + (0.0478*P*)^2^ + 0.1416*P*] where *P* = (*F*_o_^2^ + 2*F*_c_^2^)/3
6145 reflections	(Δ/σ)_max_ < 0.001
722 parameters	Δρ_max_ = 0.18 e Å^−^^3^
2 restraints	Δρ_min_ = −0.19 e Å^−^^3^

### Special details

**Table:**

Geometry. All esds (except the esd in the dihedral angle between two l.s. planes) are estimated using the full covariance matrix. The cell esds are taken into account individually in the estimation of esds in distances, angles and torsion angles; correlations between esds in cell parameters are only used when they are defined by crystal symmetry. An approximate (isotropic) treatment of cell esds is used for estimating esds involving l.s. planes.
Refinement. Refinement of *F*^2^ against ALL reflections. The weighted *R*-factor wR and goodness of fit *S* are based on *F*^2^, conventional *R*-factors *R* are based on *F*, with *F* set to zero for negative *F*^2^. The threshold expression of *F*^2^ > σ(*F*^2^) is used only for calculating *R*-factors(gt) etc. and is not relevant to the choice of reflections for refinement. *R*-factors based on *F*^2^ are statistically about twice as large as those based on *F*, and *R*- factors based on ALL data will be even larger.

### Fractional atomic coordinates and isotropic or equivalent isotropic displacement parameters (Å^2^)

**Table:**

	*x*	*y*	*z*	*U*_iso_*/*U*_eq_	
O1	0.0261 (2)	0.4566 (3)	0.46260 (13)	0.0776 (8)	
O2	0.1679 (2)	0.4288 (3)	0.53838 (12)	0.0808 (8)	
O3	0.0613 (2)	−0.2237 (3)	0.39419 (13)	0.0810 (8)	
O4	0.1978 (2)	−0.2651 (3)	0.47154 (12)	0.0814 (8)	
O5	−0.0390 (4)	0.0881 (4)	0.40416 (15)	0.1298 (16)	
O6	0.0861 (4)	0.0839 (4)	0.35339 (15)	0.1206 (13)	
O7	0.5520 (2)	0.4584 (3)	0.46114 (12)	0.0697 (7)	
O8	0.6983 (2)	0.3902 (3)	0.52061 (12)	0.0825 (8)	
O9	0.5988 (2)	−0.1978 (3)	0.39002 (12)	0.0752 (8)	
O10	0.7406 (2)	−0.2761 (3)	0.44742 (12)	0.0821 (8)	
O11	0.5285 (2)	0.1248 (2)	0.41710 (10)	0.0660 (7)	
O12	0.62082 (19)	0.0972 (3)	0.34332 (11)	0.0711 (7)	
N1	0.1186 (3)	0.4350 (3)	0.47827 (14)	0.0571 (7)	
N2	0.0276 (2)	0.4744 (2)	0.20934 (12)	0.0454 (6)	
N3	−0.01869 (19)	0.2649 (2)	0.10578 (11)	0.0388 (5)	
N4	−0.00579 (18)	0.05332 (19)	0.08514 (11)	0.0376 (5)	
N5	0.0705 (2)	−0.2008 (2)	0.14601 (12)	0.0444 (6)	
N6	0.1529 (2)	−0.2459 (3)	0.41122 (13)	0.0519 (7)	
N7	−0.0057 (4)	0.0868 (3)	0.35173 (16)	0.0824 (12)	
N8	0.6402 (3)	0.4212 (3)	0.46565 (14)	0.0583 (8)	
N9	0.4868 (2)	0.4884 (2)	0.20265 (11)	0.0452 (6)	
N10	0.42653 (18)	0.27230 (19)	0.10836 (11)	0.0360 (5)	
N11	0.43561 (19)	0.0591 (2)	0.08579 (11)	0.0404 (5)	
N12	0.5201 (3)	−0.1971 (2)	0.13218 (12)	0.0509 (7)	
N13	0.6844 (2)	−0.2400 (3)	0.39346 (13)	0.0514 (7)	
N14	0.5375 (2)	0.1073 (2)	0.35797 (12)	0.0478 (6)	
C1	0.1207 (2)	0.4378 (3)	0.35527 (15)	0.0446 (7)	
H1	0.0503	0.4625	0.3448	0.053*	
C2	0.1723 (3)	0.4170 (3)	0.42276 (16)	0.0468 (7)	
C3	0.2741 (3)	0.3794 (3)	0.44019 (18)	0.0591 (9)	
H3	0.3083	0.3649	0.4871	0.071*	
C4	0.3250 (3)	0.3634 (3)	0.3877 (2)	0.0678 (10)	
H4	0.3951	0.3370	0.3985	0.081*	
C5	0.2750 (3)	0.3854 (3)	0.31987 (18)	0.0582 (9)	
H5	0.3115	0.3751	0.2844	0.070*	
C6	0.1723 (2)	0.4224 (3)	0.30228 (15)	0.0452 (7)	
C7	0.1219 (3)	0.4467 (3)	0.22959 (15)	0.0464 (7)	
H7	0.1622	0.4414	0.1960	0.056*	
C8	−0.0121 (3)	0.4944 (3)	0.13518 (15)	0.0483 (7)	
H8A	0.0461	0.5042	0.1125	0.058*	
H8B	−0.0535	0.5741	0.1274	0.058*	
C9	−0.0786 (2)	0.3817 (3)	0.10338 (15)	0.0452 (7)	
H9A	−0.1335	0.3681	0.1287	0.054*	
H9B	−0.1121	0.4018	0.0546	0.054*	
C10	0.0469 (2)	0.2563 (3)	0.05557 (14)	0.0429 (7)	
H10A	0.1189	0.2800	0.0774	0.051*	
H10B	0.0210	0.3133	0.0154	0.051*	
C11	0.0395 (2)	0.1166 (2)	0.03351 (14)	0.0422 (7)	
H11A	−0.0052	0.1066	−0.0136	0.051*	
H11B	0.1085	0.0811	0.0341	0.051*	
C12	−0.0516 (2)	−0.0713 (3)	0.06384 (14)	0.0431 (7)	
H12A	−0.0892	−0.0671	0.0146	0.052*	
H12B	−0.1021	−0.0922	0.0917	0.052*	
C13	0.0282 (3)	−0.1764 (3)	0.07264 (14)	0.0469 (7)	
H13A	−0.0037	−0.2557	0.0498	0.056*	
H13B	0.0843	−0.1507	0.0504	0.056*	
C14	0.1664 (3)	−0.2193 (3)	0.16574 (15)	0.0450 (7)	
H14	0.2071	−0.2177	0.1324	0.054*	
C15	0.2170 (2)	−0.2432 (3)	0.23866 (15)	0.0431 (7)	
C16	0.3208 (3)	−0.2737 (3)	0.25746 (18)	0.0552 (8)	
H16	0.3593	−0.2799	0.2229	0.066*	
C17	0.3691 (3)	−0.2950 (3)	0.3262 (2)	0.0651 (10)	
H17	0.4402	−0.3163	0.3382	0.078*	
C18	0.3153 (3)	−0.2857 (3)	0.37732 (17)	0.0538 (8)	
H18	0.3482	−0.3005	0.4245	0.065*	
C19	0.2116 (2)	−0.2541 (3)	0.35782 (15)	0.0434 (7)	
C20	0.1617 (2)	−0.2336 (2)	0.29012 (14)	0.0391 (6)	
H20	0.0904	−0.2132	0.2784	0.047*	
C21	−0.0790 (2)	0.1478 (2)	0.09801 (14)	0.0389 (6)	
H21	−0.1387	0.1545	0.0570	0.047*	
C22	−0.1167 (3)	0.1193 (3)	0.16267 (15)	0.0455 (7)	
C23	−0.2200 (3)	0.0999 (3)	0.1606 (2)	0.0587 (9)	
H23	−0.2690	0.1038	0.1175	0.070*	
C24	−0.2533 (3)	0.0747 (3)	0.2207 (2)	0.0729 (13)	
H24	−0.3244	0.0613	0.2184	0.088*	
C25	−0.1831 (4)	0.0692 (3)	0.2832 (2)	0.0747 (13)	
H25	−0.2048	0.0527	0.3247	0.090*	
C26	−0.0809 (3)	0.0882 (3)	0.28462 (17)	0.0608 (10)	
C27	−0.0458 (3)	0.1124 (3)	0.22580 (16)	0.0518 (8)	
H27	0.0257	0.1241	0.2285	0.062*	
C28	0.6116 (2)	0.4386 (3)	0.33961 (14)	0.0426 (7)	
H28	0.5416	0.4593	0.3375	0.051*	
C29	0.6790 (3)	0.4144 (3)	0.40212 (15)	0.0467 (7)	
C30	0.7810 (3)	0.3836 (3)	0.40797 (19)	0.0586 (9)	
H30	0.8256	0.3683	0.4520	0.070*	
C31	0.8168 (3)	0.3758 (3)	0.3483 (2)	0.0625 (9)	
H31	0.8867	0.3537	0.3509	0.075*	
C32	0.7508 (3)	0.3999 (3)	0.28452 (19)	0.0567 (8)	
H32	0.7763	0.3943	0.2437	0.068*	
C33	0.6485 (2)	0.4322 (3)	0.27917 (15)	0.0427 (7)	
C34	0.5801 (3)	0.4583 (3)	0.21081 (14)	0.0455 (7)	
H34	0.6079	0.4520	0.1710	0.055*	
C35	0.4290 (3)	0.5037 (3)	0.13124 (14)	0.0475 (7)	
H35A	0.4773	0.5162	0.1007	0.057*	
H35B	0.3842	0.5803	0.1273	0.057*	
C36	0.3637 (2)	0.3855 (2)	0.10880 (15)	0.0426 (7)	
H36A	0.3177	0.3717	0.1408	0.051*	
H36B	0.3199	0.3995	0.0618	0.051*	
C37	0.4791 (3)	0.2630 (3)	0.05098 (14)	0.0453 (7)	
H37A	0.5527	0.2866	0.0668	0.054*	
H37B	0.4463	0.3202	0.0123	0.054*	
C38	0.4677 (3)	0.1237 (3)	0.02864 (14)	0.0466 (7)	
H38A	0.4149	0.1140	−0.0152	0.056*	
H38B	0.5340	0.0888	0.0224	0.056*	
C39	0.3833 (3)	−0.0623 (3)	0.06658 (15)	0.0523 (8)	
H39A	0.3348	−0.0532	0.0208	0.063*	
H39B	0.3426	−0.0843	0.1008	0.063*	
C40	0.4574 (3)	−0.1695 (3)	0.06335 (15)	0.0596 (9)	
H40A	0.4186	−0.2472	0.0441	0.072*	
H40B	0.5026	−0.1450	0.0323	0.072*	
C41	0.6160 (3)	−0.2077 (3)	0.13940 (15)	0.0516 (8)	
H41	0.6438	−0.1978	0.0998	0.062*	
C42	0.6867 (3)	−0.2344 (3)	0.20633 (15)	0.0462 (7)	
C43	0.7885 (3)	−0.2686 (3)	0.21040 (19)	0.0580 (9)	
H43	0.8125	−0.2748	0.1691	0.070*	
C44	0.8556 (3)	−0.2937 (3)	0.2732 (2)	0.0627 (9)	
H44	0.9247	−0.3177	0.2746	0.075*	
C45	0.8224 (3)	−0.2840 (3)	0.33372 (18)	0.0551 (8)	
H45	0.8679	−0.3005	0.3773	0.066*	
C46	0.7211 (2)	−0.2496 (3)	0.32922 (15)	0.0434 (7)	
C47	0.6526 (2)	−0.2248 (2)	0.26753 (14)	0.0419 (7)	
H47	0.5833	−0.2017	0.2664	0.050*	
C48	0.3706 (2)	0.1528 (2)	0.11034 (13)	0.0386 (6)	
H48	0.3009	0.1570	0.0781	0.046*	
C49	0.3613 (2)	0.1228 (2)	0.18271 (14)	0.0391 (6)	
C50	0.2689 (3)	0.0929 (3)	0.19902 (16)	0.0458 (7)	
H50	0.2077	0.0904	0.1632	0.055*	
C51	0.2637 (3)	0.0662 (3)	0.26671 (17)	0.0511 (8)	
H51	0.1993	0.0457	0.2767	0.061*	
C52	0.3514 (3)	0.0695 (3)	0.31922 (15)	0.0471 (7)	
H52	0.3485	0.0522	0.3657	0.056*	
C53	0.4438 (2)	0.0987 (3)	0.30269 (14)	0.0403 (6)	
C54	0.4506 (2)	0.1249 (3)	0.23579 (14)	0.0407 (6)	
H54	0.5153	0.1442	0.2260	0.049*	

### Atomic displacement parameters (Å^2^)

**Table:**

	*U*^11^	*U*^22^	*U*^33^	*U*^12^	*U*^13^	*U*^23^
O1	0.0682 (19)	0.111 (2)	0.0588 (16)	−0.0063 (16)	0.0245 (15)	−0.0097 (14)
O2	0.103 (2)	0.098 (2)	0.0386 (14)	−0.0036 (16)	0.0100 (13)	0.0131 (13)
O3	0.0531 (17)	0.148 (3)	0.0426 (14)	−0.0008 (17)	0.0129 (12)	0.0006 (15)
O4	0.087 (2)	0.118 (2)	0.0335 (13)	−0.0012 (17)	0.0018 (13)	0.0044 (13)
O5	0.209 (4)	0.149 (3)	0.0532 (17)	0.081 (3)	0.074 (2)	0.0374 (19)
O6	0.130 (3)	0.195 (4)	0.0390 (16)	0.005 (3)	0.025 (2)	0.0121 (19)
O7	0.082 (2)	0.0833 (18)	0.0458 (13)	−0.0087 (15)	0.0182 (14)	−0.0057 (12)
O8	0.094 (2)	0.102 (2)	0.0379 (13)	−0.0326 (17)	−0.0144 (13)	0.0177 (13)
O9	0.0601 (17)	0.125 (2)	0.0423 (13)	0.0025 (16)	0.0165 (13)	−0.0044 (13)
O10	0.084 (2)	0.117 (2)	0.0357 (13)	0.0067 (17)	−0.0046 (13)	0.0056 (13)
O11	0.0762 (17)	0.0962 (18)	0.0251 (11)	0.0074 (14)	0.0111 (11)	0.0000 (11)
O12	0.0516 (16)	0.120 (2)	0.0399 (13)	0.0023 (14)	0.0069 (11)	0.0042 (13)
N1	0.074 (2)	0.0565 (16)	0.0415 (16)	−0.0090 (15)	0.0150 (15)	0.0024 (12)
N2	0.0597 (18)	0.0381 (12)	0.0393 (13)	−0.0023 (12)	0.0135 (12)	−0.0042 (10)
N3	0.0482 (15)	0.0378 (12)	0.0306 (12)	−0.0011 (10)	0.0097 (11)	0.0013 (9)
N4	0.0485 (15)	0.0373 (11)	0.0277 (11)	−0.0026 (10)	0.0105 (10)	0.0007 (9)
N5	0.0650 (19)	0.0387 (12)	0.0307 (12)	0.0001 (12)	0.0135 (12)	0.0005 (10)
N6	0.061 (2)	0.0648 (16)	0.0272 (13)	−0.0085 (13)	0.0037 (13)	0.0000 (11)
N7	0.137 (4)	0.079 (2)	0.0431 (18)	0.030 (2)	0.046 (2)	0.0179 (15)
N8	0.075 (2)	0.0571 (16)	0.0353 (15)	−0.0220 (16)	−0.0019 (15)	0.0034 (12)
N9	0.0639 (19)	0.0375 (12)	0.0302 (12)	0.0017 (12)	0.0026 (12)	−0.0027 (9)
N10	0.0453 (14)	0.0339 (11)	0.0288 (11)	−0.0003 (10)	0.0082 (10)	0.0015 (9)
N11	0.0560 (16)	0.0363 (12)	0.0268 (11)	−0.0016 (10)	0.0053 (11)	−0.0001 (9)
N12	0.082 (2)	0.0359 (12)	0.0296 (13)	0.0015 (13)	0.0015 (13)	−0.0004 (10)
N13	0.0582 (19)	0.0609 (16)	0.0328 (14)	−0.0116 (14)	0.0062 (13)	−0.0036 (11)
N14	0.0582 (18)	0.0567 (15)	0.0273 (12)	0.0035 (13)	0.0076 (12)	0.0053 (10)
C1	0.0492 (19)	0.0394 (14)	0.0445 (17)	−0.0036 (13)	0.0096 (15)	−0.0016 (12)
C2	0.055 (2)	0.0399 (14)	0.0424 (17)	−0.0038 (14)	0.0062 (15)	−0.0009 (12)
C3	0.062 (2)	0.0527 (18)	0.054 (2)	0.0063 (16)	−0.0031 (18)	−0.0061 (15)
C4	0.054 (2)	0.072 (2)	0.068 (2)	0.0124 (18)	−0.0045 (19)	−0.0197 (19)
C5	0.051 (2)	0.062 (2)	0.061 (2)	0.0056 (16)	0.0131 (18)	−0.0193 (16)
C6	0.0486 (19)	0.0409 (15)	0.0455 (17)	−0.0025 (13)	0.0096 (15)	−0.0095 (12)
C7	0.054 (2)	0.0446 (16)	0.0431 (17)	−0.0017 (14)	0.0154 (15)	−0.0100 (13)
C8	0.067 (2)	0.0389 (15)	0.0386 (15)	−0.0011 (14)	0.0109 (15)	−0.0003 (12)
C9	0.0540 (19)	0.0409 (14)	0.0390 (15)	0.0029 (13)	0.0071 (14)	0.0024 (12)
C10	0.0529 (18)	0.0433 (15)	0.0337 (15)	−0.0043 (13)	0.0125 (14)	0.0033 (11)
C11	0.0545 (18)	0.0463 (15)	0.0265 (13)	−0.0055 (13)	0.0109 (13)	0.0023 (11)
C12	0.059 (2)	0.0379 (14)	0.0316 (14)	−0.0072 (13)	0.0087 (14)	−0.0023 (11)
C13	0.067 (2)	0.0418 (15)	0.0312 (15)	−0.0001 (14)	0.0108 (14)	−0.0014 (12)
C14	0.063 (2)	0.0393 (15)	0.0368 (16)	−0.0051 (14)	0.0202 (16)	−0.0046 (12)
C15	0.0499 (19)	0.0379 (14)	0.0426 (16)	−0.0072 (13)	0.0130 (14)	−0.0069 (12)
C16	0.049 (2)	0.0596 (19)	0.060 (2)	−0.0045 (15)	0.0190 (17)	−0.0140 (16)
C17	0.047 (2)	0.072 (2)	0.070 (3)	0.0045 (17)	0.0008 (19)	−0.0147 (19)
C18	0.049 (2)	0.0567 (18)	0.0471 (18)	−0.0007 (15)	−0.0061 (16)	−0.0064 (15)
C19	0.0501 (19)	0.0406 (14)	0.0367 (15)	−0.0047 (12)	0.0045 (14)	−0.0023 (12)
C20	0.0411 (17)	0.0402 (14)	0.0349 (14)	−0.0060 (12)	0.0063 (13)	−0.0030 (11)
C21	0.0405 (16)	0.0405 (14)	0.0344 (14)	−0.0026 (12)	0.0062 (12)	0.0007 (11)
C22	0.056 (2)	0.0380 (14)	0.0478 (18)	−0.0014 (13)	0.0228 (16)	−0.0020 (12)
C23	0.058 (2)	0.0480 (17)	0.078 (2)	−0.0057 (15)	0.0318 (19)	−0.0136 (16)
C24	0.081 (3)	0.0456 (19)	0.113 (4)	−0.0134 (18)	0.066 (3)	−0.013 (2)
C25	0.115 (4)	0.0456 (19)	0.088 (3)	0.000 (2)	0.075 (3)	0.0001 (19)
C26	0.100 (3)	0.0452 (16)	0.0485 (19)	0.0045 (18)	0.040 (2)	0.0065 (14)
C27	0.066 (2)	0.0502 (17)	0.0465 (18)	0.0026 (15)	0.0283 (17)	0.0080 (14)
C28	0.0518 (19)	0.0382 (14)	0.0344 (15)	−0.0036 (13)	0.0033 (14)	−0.0009 (11)
C29	0.059 (2)	0.0381 (14)	0.0379 (16)	−0.0087 (13)	0.0005 (15)	0.0032 (12)
C30	0.055 (2)	0.0504 (18)	0.060 (2)	−0.0016 (16)	−0.0089 (17)	0.0058 (15)
C31	0.047 (2)	0.0567 (19)	0.077 (3)	0.0045 (15)	0.0008 (19)	−0.0090 (17)
C32	0.057 (2)	0.0535 (18)	0.060 (2)	−0.0013 (16)	0.0138 (18)	−0.0132 (15)
C33	0.0500 (19)	0.0355 (14)	0.0397 (16)	−0.0040 (13)	0.0044 (14)	−0.0048 (12)
C34	0.063 (2)	0.0397 (15)	0.0333 (15)	−0.0055 (14)	0.0110 (15)	−0.0054 (11)
C35	0.071 (2)	0.0360 (14)	0.0319 (15)	0.0048 (14)	0.0040 (15)	0.0004 (11)
C36	0.0516 (18)	0.0405 (14)	0.0317 (14)	0.0064 (13)	0.0020 (13)	0.0020 (11)
C37	0.061 (2)	0.0443 (15)	0.0319 (14)	−0.0033 (14)	0.0144 (14)	−0.0001 (12)
C38	0.066 (2)	0.0448 (15)	0.0292 (14)	0.0018 (14)	0.0105 (14)	0.0006 (11)
C39	0.076 (2)	0.0385 (15)	0.0342 (16)	−0.0087 (14)	−0.0032 (15)	−0.0003 (12)
C40	0.102 (3)	0.0370 (15)	0.0324 (16)	0.0032 (16)	−0.0001 (16)	−0.0030 (12)
C41	0.087 (3)	0.0377 (15)	0.0330 (16)	−0.0007 (16)	0.0198 (17)	−0.0057 (12)
C42	0.064 (2)	0.0359 (14)	0.0408 (16)	−0.0062 (14)	0.0157 (15)	−0.0038 (12)
C43	0.069 (2)	0.0528 (18)	0.061 (2)	−0.0030 (17)	0.033 (2)	−0.0074 (15)
C44	0.053 (2)	0.066 (2)	0.070 (2)	−0.0019 (17)	0.018 (2)	−0.0092 (18)
C45	0.047 (2)	0.0577 (19)	0.056 (2)	−0.0038 (15)	0.0020 (16)	−0.0058 (15)
C46	0.0480 (19)	0.0443 (15)	0.0374 (15)	−0.0059 (13)	0.0091 (14)	−0.0031 (12)
C47	0.0514 (19)	0.0381 (14)	0.0357 (15)	−0.0047 (13)	0.0088 (14)	−0.0039 (12)
C48	0.0445 (17)	0.0394 (14)	0.0291 (13)	−0.0012 (12)	0.0026 (12)	0.0025 (11)
C49	0.0465 (18)	0.0362 (13)	0.0329 (14)	−0.0027 (12)	0.0059 (13)	0.0016 (11)
C50	0.0500 (19)	0.0424 (15)	0.0424 (16)	−0.0086 (13)	0.0053 (14)	0.0029 (12)
C51	0.056 (2)	0.0504 (17)	0.0495 (18)	−0.0110 (15)	0.0170 (16)	0.0057 (14)
C52	0.063 (2)	0.0436 (15)	0.0376 (16)	−0.0042 (14)	0.0187 (16)	0.0046 (12)
C53	0.0504 (18)	0.0394 (14)	0.0302 (14)	0.0002 (13)	0.0076 (13)	0.0005 (11)
C54	0.0494 (18)	0.0433 (15)	0.0297 (14)	−0.0001 (13)	0.0098 (13)	0.0018 (11)

### Geometric parameters (Å, °)

**Table:**

O1—N1	1.222 (4)	C16—H16	0.9500
O2—N1	1.223 (4)	C17—C18	1.375 (5)
O3—N6	1.213 (4)	C17—H17	0.9500
O4—N6	1.224 (3)	C18—C19	1.387 (5)
O5—N7	1.223 (4)	C18—H18	0.9500
O6—N7	1.218 (5)	C19—C20	1.371 (4)
O7—N8	1.223 (4)	C20—H20	0.9500
O8—N8	1.226 (4)	C21—C22	1.513 (4)
O9—N13	1.212 (4)	C21—H21	1.0000
O10—N13	1.215 (4)	C22—C23	1.384 (5)
O11—N14	1.221 (3)	C22—C27	1.386 (5)
O12—N14	1.217 (3)	C23—C24	1.390 (5)
N1—C2	1.459 (4)	C23—H23	0.9500
N2—C7	1.262 (4)	C24—C25	1.371 (6)
N2—C8	1.459 (4)	C24—H24	0.9500
N3—C21	1.451 (3)	C25—C26	1.371 (6)
N3—C9	1.453 (3)	C25—H25	0.9500
N3—C10	1.474 (4)	C26—C27	1.379 (4)
N4—C21	1.452 (3)	C27—H27	0.9500
N4—C12	1.458 (3)	C28—C29	1.376 (4)
N4—C11	1.462 (3)	C28—C33	1.400 (4)
N5—C14	1.263 (4)	C28—H28	0.9500
N5—C13	1.457 (4)	C29—C30	1.377 (5)
N6—C19	1.459 (4)	C30—C31	1.378 (5)
N7—C26	1.470 (5)	C30—H30	0.9500
N8—C29	1.472 (4)	C31—C32	1.387 (5)
N9—C34	1.258 (4)	C31—H31	0.9500
N9—C35	1.456 (4)	C32—C33	1.386 (4)
N10—C36	1.450 (3)	C32—H32	0.9500
N10—C48	1.459 (3)	C33—C34	1.474 (4)
N10—C37	1.473 (3)	C34—H34	0.9500
N11—C39	1.454 (3)	C35—C36	1.516 (4)
N11—C48	1.463 (4)	C35—H35A	0.9900
N11—C38	1.467 (3)	C35—H35B	0.9900
N12—C41	1.260 (4)	C36—H36A	0.9900
N12—C40	1.455 (4)	C36—H36B	0.9900
N13—C46	1.472 (4)	C37—C38	1.517 (4)
N14—C53	1.462 (4)	C37—H37A	0.9900
C1—C2	1.373 (4)	C37—H37B	0.9900
C1—C6	1.394 (4)	C38—H38A	0.9900
C1—H1	0.9500	C38—H38B	0.9900
C2—C3	1.378 (5)	C39—C40	1.504 (5)
C3—C4	1.380 (5)	C39—H39A	0.9900
C3—H3	0.9500	C39—H39B	0.9900
C4—C5	1.376 (5)	C40—H40A	0.9900
C4—H4	0.9500	C40—H40B	0.9900
C5—C6	1.388 (4)	C41—C42	1.466 (5)
C5—H5	0.9500	C41—H41	0.9500
C6—C7	1.465 (4)	C42—C43	1.388 (5)
C7—H7	0.9500	C42—C47	1.397 (4)
C8—C9	1.519 (4)	C43—C44	1.379 (5)
C8—H8A	0.9900	C43—H43	0.9500
C8—H8B	0.9900	C44—C45	1.378 (5)
C9—H9A	0.9900	C44—H44	0.9500
C9—H9B	0.9900	C45—C46	1.381 (5)
C10—C11	1.519 (4)	C45—H45	0.9500
C10—H10A	0.9900	C46—C47	1.372 (4)
C10—H10B	0.9900	C47—H47	0.9500
C11—H11A	0.9900	C48—C49	1.503 (4)
C11—H11B	0.9900	C48—H48	1.0000
C12—C13	1.510 (4)	C49—C50	1.381 (4)
C12—H12A	0.9900	C49—C54	1.395 (4)
C12—H12B	0.9900	C50—C51	1.390 (4)
C13—H13A	0.9900	C50—H50	0.9500
C13—H13B	0.9900	C51—C52	1.374 (5)
C14—C15	1.469 (4)	C51—H51	0.9500
C14—H14	0.9500	C52—C53	1.382 (4)
C15—C16	1.385 (4)	C52—H52	0.9500
C15—C20	1.396 (4)	C53—C54	1.379 (3)
C16—C17	1.385 (5)	C54—H54	0.9500
			
O1—N1—O2	122.7 (3)	C23—C22—C21	122.0 (3)
O1—N1—C2	118.4 (3)	C27—C22—C21	119.0 (3)
O2—N1—C2	118.9 (3)	C22—C23—C24	121.1 (4)
C7—N2—C8	117.1 (3)	C22—C23—H23	119.4
C21—N3—C9	114.6 (2)	C24—C23—H23	119.4
C21—N3—C10	106.2 (2)	C25—C24—C23	119.8 (4)
C9—N3—C10	115.9 (2)	C25—C24—H24	120.1
C21—N4—C12	113.8 (2)	C23—C24—H24	120.1
C21—N4—C11	102.8 (2)	C24—C25—C26	118.6 (3)
C12—N4—C11	114.4 (2)	C24—C25—H25	120.7
C14—N5—C13	117.7 (3)	C26—C25—H25	120.7
O3—N6—O4	122.4 (3)	C25—C26—C27	122.8 (4)
O3—N6—C19	119.0 (3)	C25—C26—N7	118.9 (3)
O4—N6—C19	118.6 (3)	C27—C26—N7	118.3 (4)
O6—N7—O5	122.6 (4)	C26—C27—C22	118.7 (3)
O6—N7—C26	119.7 (3)	C26—C27—H27	120.7
O5—N7—C26	117.7 (5)	C22—C27—H27	120.7
O7—N8—O8	123.2 (3)	C29—C28—C33	118.6 (3)
O7—N8—C29	118.5 (3)	C29—C28—H28	120.7
O8—N8—C29	118.3 (3)	C33—C28—H28	120.7
C34—N9—C35	115.9 (2)	C28—C29—C30	123.0 (3)
C36—N10—C48	113.4 (2)	C28—C29—N8	118.6 (3)
C36—N10—C37	116.0 (2)	C30—C29—N8	118.5 (3)
C48—N10—C37	107.65 (19)	C29—C30—C31	118.3 (3)
C39—N11—C48	112.6 (2)	C29—C30—H30	120.8
C39—N11—C38	114.0 (2)	C31—C30—H30	120.8
C48—N11—C38	104.7 (2)	C30—C31—C32	120.0 (3)
C41—N12—C40	118.1 (3)	C30—C31—H31	120.0
O9—N13—O10	122.8 (3)	C32—C31—H31	120.0
O9—N13—C46	118.3 (3)	C33—C32—C31	121.3 (3)
O10—N13—C46	118.8 (3)	C33—C32—H32	119.4
O12—N14—O11	122.8 (3)	C31—C32—H32	119.4
O12—N14—C53	119.0 (2)	C32—C33—C28	118.8 (3)
O11—N14—C53	118.2 (3)	C32—C33—C34	120.2 (3)
C2—C1—C6	119.7 (3)	C28—C33—C34	121.0 (3)
C2—C1—H1	120.2	N9—C34—C33	123.1 (3)
C6—C1—H1	120.2	N9—C34—H34	118.4
C1—C2—C3	122.0 (3)	C33—C34—H34	118.4
C1—C2—N1	119.6 (3)	N9—C35—C36	109.5 (2)
C3—C2—N1	118.4 (3)	N9—C35—H35A	109.8
C2—C3—C4	118.3 (3)	C36—C35—H35A	109.8
C2—C3—H3	120.8	N9—C35—H35B	109.8
C4—C3—H3	120.8	C36—C35—H35B	109.8
C5—C4—C3	120.5 (3)	H35A—C35—H35B	108.2
C5—C4—H4	119.8	N10—C36—C35	111.8 (2)
C3—C4—H4	119.8	N10—C36—H36A	109.3
C4—C5—C6	121.2 (3)	C35—C36—H36A	109.3
C4—C5—H5	119.4	N10—C36—H36B	109.3
C6—C5—H5	119.4	C35—C36—H36B	109.3
C5—C6—C1	118.3 (3)	H36A—C36—H36B	107.9
C5—C6—C7	119.7 (3)	N10—C37—C38	104.8 (2)
C1—C6—C7	121.9 (3)	N10—C37—H37A	110.8
N2—C7—C6	123.3 (3)	C38—C37—H37A	110.8
N2—C7—H7	118.4	N10—C37—H37B	110.8
C6—C7—H7	118.4	C38—C37—H37B	110.8
N2—C8—C9	110.4 (2)	H37A—C37—H37B	108.9
N2—C8—H8A	109.6	N11—C38—C37	104.0 (2)
C9—C8—H8A	109.6	N11—C38—H38A	111.0
N2—C8—H8B	109.6	C37—C38—H38A	111.0
C9—C8—H8B	109.6	N11—C38—H38B	111.0
H8A—C8—H8B	108.1	C37—C38—H38B	111.0
N3—C9—C8	111.9 (3)	H38A—C38—H38B	109.0
N3—C9—H9A	109.2	N11—C39—C40	112.3 (3)
C8—C9—H9A	109.2	N11—C39—H39A	109.1
N3—C9—H9B	109.2	C40—C39—H39A	109.1
C8—C9—H9B	109.2	N11—C39—H39B	109.1
H9A—C9—H9B	107.9	C40—C39—H39B	109.1
N3—C10—C11	104.1 (2)	H39A—C39—H39B	107.9
N3—C10—H10A	110.9	N12—C40—C39	110.3 (2)
C11—C10—H10A	110.9	N12—C40—H40A	109.6
N3—C10—H10B	110.9	C39—C40—H40A	109.6
C11—C10—H10B	110.9	N12—C40—H40B	109.6
H10A—C10—H10B	109.0	C39—C40—H40B	109.6
N4—C11—C10	103.9 (2)	H40A—C40—H40B	108.1
N4—C11—H11A	111.0	N12—C41—C42	122.8 (3)
C10—C11—H11A	111.0	N12—C41—H41	118.6
N4—C11—H11B	111.0	C42—C41—H41	118.6
C10—C11—H11B	111.0	C43—C42—C47	118.7 (3)
H11A—C11—H11B	109.0	C43—C42—C41	121.1 (3)
N4—C12—C13	112.2 (2)	C47—C42—C41	120.2 (3)
N4—C12—H12A	109.2	C44—C43—C42	121.4 (3)
C13—C12—H12A	109.2	C44—C43—H43	119.3
N4—C12—H12B	109.2	C42—C43—H43	119.3
C13—C12—H12B	109.2	C45—C44—C43	120.1 (3)
H12A—C12—H12B	107.9	C45—C44—H44	119.9
N5—C13—C12	109.9 (2)	C43—C44—H44	119.9
N5—C13—H13A	109.7	C44—C45—C46	118.1 (3)
C12—C13—H13A	109.7	C44—C45—H45	121.0
N5—C13—H13B	109.7	C46—C45—H45	121.0
C12—C13—H13B	109.7	C47—C46—C45	123.1 (3)
H13A—C13—H13B	108.2	C47—C46—N13	118.3 (3)
N5—C14—C15	122.1 (3)	C45—C46—N13	118.6 (3)
N5—C14—H14	118.9	C46—C47—C42	118.6 (3)
C15—C14—H14	118.9	C46—C47—H47	120.7
C16—C15—C20	119.0 (3)	C42—C47—H47	120.7
C16—C15—C14	120.4 (3)	N10—C48—N11	102.4 (2)
C20—C15—C14	120.6 (3)	N10—C48—C49	111.1 (2)
C17—C16—C15	120.6 (3)	N11—C48—C49	111.5 (2)
C17—C16—H16	119.7	N10—C48—H48	110.5
C15—C16—H16	119.7	N11—C48—H48	110.5
C18—C17—C16	120.9 (3)	C49—C48—H48	110.5
C18—C17—H17	119.6	C50—C49—C54	118.6 (3)
C16—C17—H17	119.6	C50—C49—C48	123.2 (3)
C17—C18—C19	117.9 (3)	C54—C49—C48	118.2 (2)
C17—C18—H18	121.0	C49—C50—C51	121.3 (3)
C19—C18—H18	121.0	C49—C50—H50	119.3
C20—C19—C18	122.4 (3)	C51—C50—H50	119.3
C20—C19—N6	118.9 (3)	C52—C51—C50	120.2 (3)
C18—C19—N6	118.7 (3)	C52—C51—H51	119.9
C19—C20—C15	119.2 (3)	C50—C51—H51	119.9
C19—C20—H20	120.4	C51—C52—C53	118.3 (3)
C15—C20—H20	120.4	C51—C52—H52	120.8
N3—C21—N4	102.2 (2)	C53—C52—H52	120.8
N3—C21—C22	111.2 (2)	C54—C53—C52	122.3 (3)
N4—C21—C22	112.7 (2)	C54—C53—N14	118.3 (2)
N3—C21—H21	110.2	C52—C53—N14	119.3 (2)
N4—C21—H21	110.2	C53—C54—C49	119.3 (3)
C22—C21—H21	110.2	C53—C54—H54	120.4
C23—C22—C27	119.0 (3)	C49—C54—H54	120.4
			
C6—C1—C2—C3	−0.8 (4)	C33—C28—C29—C30	−0.2 (4)
C6—C1—C2—N1	179.1 (3)	C33—C28—C29—N8	180.0 (2)
O1—N1—C2—C1	6.8 (4)	O7—N8—C29—C28	−5.9 (4)
O2—N1—C2—C1	−172.7 (3)	O8—N8—C29—C28	174.6 (3)
O1—N1—C2—C3	−173.3 (3)	O7—N8—C29—C30	174.3 (3)
O2—N1—C2—C3	7.2 (4)	O8—N8—C29—C30	−5.2 (4)
C1—C2—C3—C4	0.4 (5)	C28—C29—C30—C31	−0.6 (5)
N1—C2—C3—C4	−179.5 (3)	N8—C29—C30—C31	179.2 (3)
C2—C3—C4—C5	0.4 (5)	C29—C30—C31—C32	0.7 (5)
C3—C4—C5—C6	−0.9 (5)	C30—C31—C32—C33	0.0 (5)
C4—C5—C6—C1	0.4 (5)	C31—C32—C33—C28	−0.8 (5)
C4—C5—C6—C7	179.2 (3)	C31—C32—C33—C34	179.6 (3)
C2—C1—C6—C5	0.4 (4)	C29—C28—C33—C32	0.9 (4)
C2—C1—C6—C7	−178.3 (3)	C29—C28—C33—C34	−179.5 (3)
C8—N2—C7—C6	−179.2 (2)	C35—N9—C34—C33	−176.7 (2)
C5—C6—C7—N2	176.4 (3)	C32—C33—C34—N9	−179.8 (3)
C1—C6—C7—N2	−5.0 (5)	C28—C33—C34—N9	0.7 (4)
C7—N2—C8—C9	106.7 (3)	C34—N9—C35—C36	101.8 (3)
C21—N3—C9—C8	160.7 (2)	C48—N10—C36—C35	160.4 (2)
C10—N3—C9—C8	−75.0 (3)	C37—N10—C36—C35	−74.3 (3)
N2—C8—C9—N3	−66.2 (3)	N9—C35—C36—N10	−64.0 (3)
C21—N3—C10—C11	−13.9 (3)	C36—N10—C37—C38	−138.6 (3)
C9—N3—C10—C11	−142.4 (2)	C48—N10—C37—C38	−10.4 (3)
C21—N4—C11—C10	37.1 (3)	C39—N11—C38—C37	158.1 (3)
C12—N4—C11—C10	161.0 (2)	C48—N11—C38—C37	34.7 (3)
N3—C10—C11—N4	−14.3 (3)	N10—C37—C38—N11	−14.8 (3)
C21—N4—C12—C13	−162.0 (2)	C48—N11—C39—C40	−161.8 (2)
C11—N4—C12—C13	80.3 (3)	C38—N11—C39—C40	79.2 (3)
C14—N5—C13—C12	−138.7 (3)	C41—N12—C40—C39	−132.4 (3)
N4—C12—C13—N5	69.7 (3)	N11—C39—C40—N12	66.8 (3)
C13—N5—C14—C15	179.3 (2)	C40—N12—C41—C42	179.6 (2)
N5—C14—C15—C16	174.6 (3)	N12—C41—C42—C43	168.0 (3)
N5—C14—C15—C20	−6.4 (4)	N12—C41—C42—C47	−12.5 (4)
C20—C15—C16—C17	0.4 (4)	C47—C42—C43—C44	0.4 (4)
C14—C15—C16—C17	179.4 (3)	C41—C42—C43—C44	179.9 (3)
C15—C16—C17—C18	−0.4 (5)	C42—C43—C44—C45	−0.6 (5)
C16—C17—C18—C19	−0.1 (5)	C43—C44—C45—C46	0.4 (5)
C17—C18—C19—C20	0.7 (5)	C44—C45—C46—C47	0.0 (5)
C17—C18—C19—N6	179.0 (3)	C44—C45—C46—N13	179.5 (3)
O3—N6—C19—C20	1.4 (4)	O9—N13—C46—C47	−7.6 (4)
O4—N6—C19—C20	179.5 (3)	O10—N13—C46—C47	171.4 (3)
O3—N6—C19—C18	−176.9 (3)	O9—N13—C46—C45	172.9 (3)
O4—N6—C19—C18	1.2 (4)	O10—N13—C46—C45	−8.1 (4)
C18—C19—C20—C15	−0.8 (4)	C45—C46—C47—C42	−0.2 (4)
N6—C19—C20—C15	−179.0 (2)	N13—C46—C47—C42	−179.7 (2)
C16—C15—C20—C19	0.2 (4)	C43—C42—C47—C46	0.0 (4)
C14—C15—C20—C19	−178.8 (2)	C41—C42—C47—C46	−179.5 (2)
C9—N3—C21—N4	166.4 (2)	C36—N10—C48—N11	161.3 (2)
C10—N3—C21—N4	37.1 (3)	C37—N10—C48—N11	31.6 (3)
C9—N3—C21—C22	−73.1 (3)	C36—N10—C48—C49	−79.5 (3)
C10—N3—C21—C22	157.6 (2)	C37—N10—C48—C49	150.8 (2)
C12—N4—C21—N3	−170.3 (2)	C39—N11—C48—N10	−165.4 (2)
C11—N4—C21—N3	−46.1 (2)	C38—N11—C48—N10	−41.1 (3)
C12—N4—C21—C22	70.3 (3)	C39—N11—C48—C49	75.7 (3)
C11—N4—C21—C22	−165.5 (2)	C38—N11—C48—C49	−160.0 (2)
N3—C21—C22—C23	125.4 (3)	N10—C48—C49—C50	129.1 (3)
N4—C21—C22—C23	−120.5 (3)	N11—C48—C49—C50	−117.3 (3)
N3—C21—C22—C27	−54.5 (3)	N10—C48—C49—C54	−51.1 (3)
N4—C21—C22—C27	59.5 (3)	N11—C48—C49—C54	62.4 (3)
C27—C22—C23—C24	0.4 (4)	C54—C49—C50—C51	0.5 (4)
C21—C22—C23—C24	−179.5 (3)	C48—C49—C50—C51	−179.7 (3)
C22—C23—C24—C25	0.2 (5)	C49—C50—C51—C52	0.1 (4)
C23—C24—C25—C26	−0.4 (5)	C50—C51—C52—C53	−0.6 (4)
C24—C25—C26—C27	−0.1 (5)	C51—C52—C53—C54	0.3 (4)
C24—C25—C26—N7	178.0 (3)	C51—C52—C53—N14	177.8 (3)
O6—N7—C26—C25	168.9 (4)	O12—N14—C53—C54	−22.7 (4)
O5—N7—C26—C25	−10.9 (5)	O11—N14—C53—C54	157.1 (3)
O6—N7—C26—C27	−13.0 (6)	O12—N14—C53—C52	159.7 (3)
O5—N7—C26—C27	167.3 (3)	O11—N14—C53—C52	−20.4 (4)
C25—C26—C27—C22	0.7 (5)	C52—C53—C54—C49	0.3 (4)
N7—C26—C27—C22	−177.3 (3)	N14—C53—C54—C49	−177.2 (2)
C23—C22—C27—C26	−0.9 (4)	C50—C49—C54—C53	−0.7 (4)
C21—C22—C27—C26	179.1 (3)	C48—C49—C54—C53	179.5 (2)
